# Rapidly Progressive Infectious Pseudoaneurysm of the Ascending Aorta Caused by *Streptococcus agalactiae*: A Case Report and Literature Review

**DOI:** 10.70352/scrj.cr.26-0427

**Published:** 2026-09-05

**Authors:** Haruki Nomura, Ryohei Ushioda, Hana Fukuta, Koji Kagawa, Kaname Shimizu, Kentaro Shirakura, Yuki Setogawa, Keisuke Shibagaki, Hiroyuki Miyamoto, Shougo Takahashi, Shingo Kunioka, Hayato Ise, Mishie Tanino, Hiroyuki Kamiya

**Affiliations:** 1 Department of Cardiac Surgery, Asahikawa Medical University, Asahikawa, Hokkaido, Japan; 2 Department of Diagnostic Pathology, Asahikawa Medical University Hospital, Asahikawa, Hokkaido, Japan

**Keywords:** infectious aortic aneurysm, ascending aorta, *Streptococcus agalactiae*, pseudoaneurysm

## Abstract

**INTRODUCTION:**

Infectious aortic aneurysms are uncommon but life-threatening conditions associated with high mortality. Although they account for less than 3% of all aortic aneurysms, their clinical course may be rapidly progressive. *Streptococcus agalactiae* is a rare cause of infectious aortic aneurysm. We report a case of a rapidly progressive infectious pseudoaneurysm of the ascending aorta caused by *S. agalactiae* in an elderly woman with untreated diabetes.

**CASE PRESENTATION:**

A 77-year-old woman with untreated diabetes presented with fever and malaise and was initially diagnosed with a left renal abscess at a referring hospital. Blood cultures grew *S. agalactiae*, and intravenous antibiotics were started. Despite treatment, contrast-enhanced CT performed 6 days after admission revealed a newly developed, rapidly enlarging 65-mm pseudoaneurysm of the ascending aorta. She was urgently transferred to our institution and underwent emergency ascending aortic replacement under hypothermic circulatory arrest. Intraoperative findings revealed a saccular pseudoaneurysm containing purulent material, consistent with an infectious process. Postoperatively, she received 6 weeks of intravenous antibiotics followed by oral suppressive therapy. Her postoperative course was uneventful, and no recurrence was observed during 2 years of follow-up.

**CONCLUSIONS:**

Infectious aortic aneurysms caused by *S. agalactiae* are extremely rare but may progress rapidly despite appropriate antibiotic therapy. Patients with risk factors such as advanced age and poorly controlled diabetes may have a particularly aggressive disease course. Early CT evaluation, close clinical monitoring, and timely surgical intervention are essential for preventing catastrophic complications.

## INTRODUCTION

Infectious aortic aneurysms are rare, accounting for approximately 0.7%–2.6% of all aortic aneurysms.^1)^ Despite their low incidence, they are associated with significantly higher mortality than noninfectious aortic aneurysms.^2)^ The most commonly reported causative pathogens include *Staphylococcus* species, *Escherichia coli*, and *Salmonella* species.^3)^ *Streptococcus agalactiae* is a rare causative organism in infectious aortic aneurysm. However, infections caused by this pathogen may progress rapidly and result in extensive tissue destruction.^4)^ We report a case of a rapidly progressive infectious pseudoaneurysm of the ascending aorta caused by *S. agalactiae* that was successfully managed with surgery and antibiotic therapy. We also review previously reported cases to summarize their clinical characteristics, treatment strategies, and outcomes.

## CASE PRESENTATION

A 77-year-old woman with untreated diabetes presented with a 1-week history of fever and malaise. She was initially admitted to another hospital with a diagnosis of a left renal abscess. Laboratory tests showed elevated inflammatory markers and a hemoglobin A1c level of 13.8%. Blood cultures obtained on admission grew *S. agalactiae*, and intravenous antibiotic therapy was initiated.

**Fig. 1:**
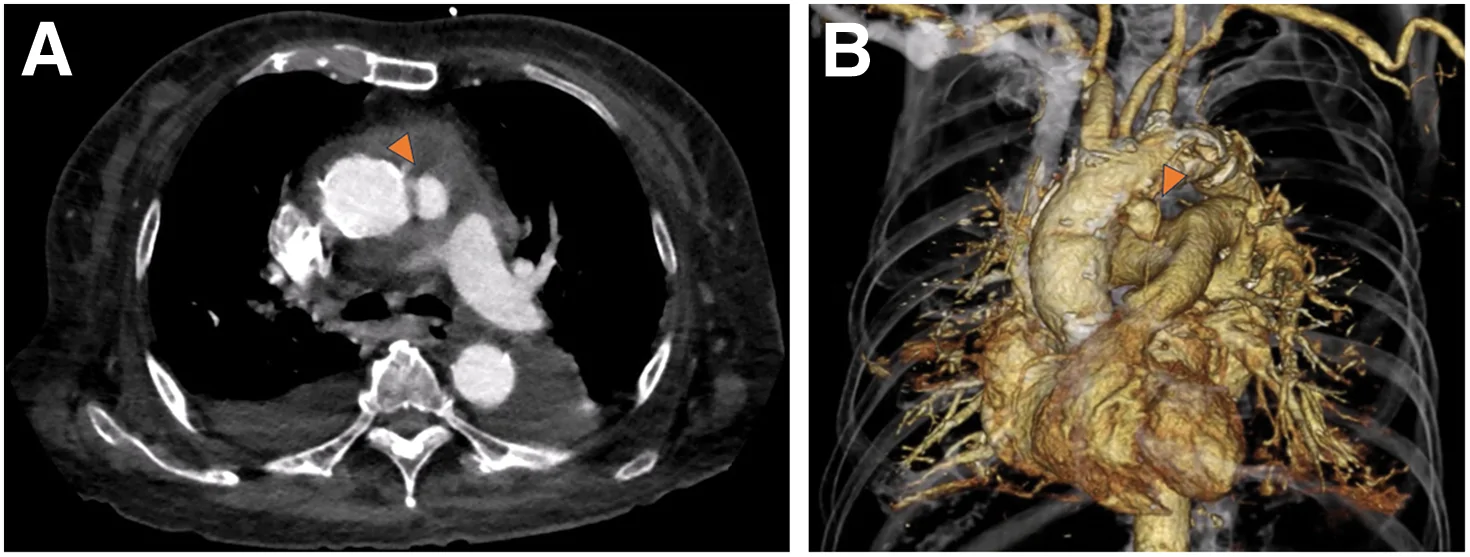
(**A**) Contrast-enhanced CT demonstrating an infectious pseudoaneurysm of the ascending aorta. (**B**) 3D CT image showing the same lesion (arrowheads).

Despite antibiotic therapy, follow-up contrast-enhanced CT performed on hospital day 6 revealed a newly developed pseudoaneurysm of the ascending aorta measuring 65 mm in diameter (**Fig. 1**). The patient was transferred to our institution, where emergency surgery was performed immediately because of the rapid enlargement of the pseudoaneurysm and the high risk of rupture.

**Fig. 2:**
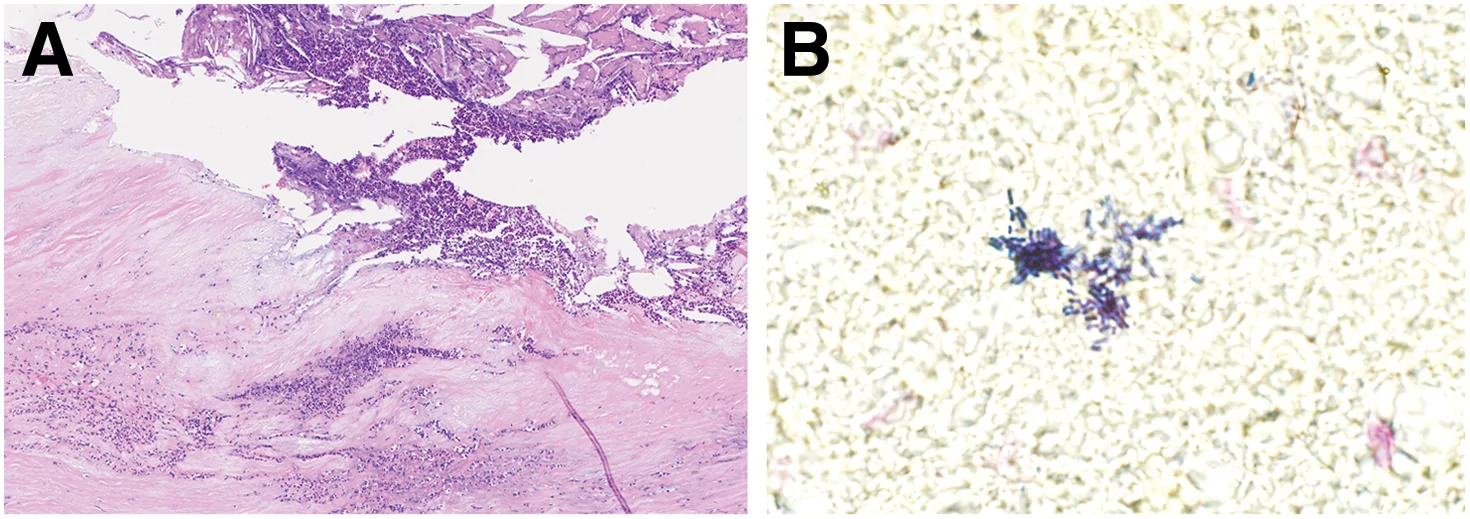
(**A**) Ascending aortic aneurysm—on the luminal side of the arterial wall, microscopically shows neutrophil infiltration. (**B**) Ascending aortic aneurysm―bacteria/tissue gram stain of the thrombus (100×, oil immersion) shows Gram positive cocci in pairs or chains consistent with Group B Streptococci.

Ascending aortic replacement was performed using a 24-mm J-Graft (Japan Lifeline, Tokyo, Japan) soaked in rifampicin. Cardiopulmonary bypass was established with arterial cannulation of the ascending aorta and venous cannulation of the right atrium. Circulatory arrest was instituted at a rectal temperature of 25.4°C. Upon opening the ascending aorta, an approximately 1-cm orifice was identified on the left lateral wall, communicating with a saccular pseudoaneurysm protruding toward the pulmonary artery. The lesion was densely adherent to the surrounding tissues, and purulent material was drained from the aneurysmal sac, consistent with an infectious process. An omental flap was not used because preoperative CT suggested pancreatic cancer. Histopathologic examination of the resected aortic wall demonstrated infiltration with Gram-positive cocci within the arterial wall (**Fig. 2**).

**Fig. 3:**
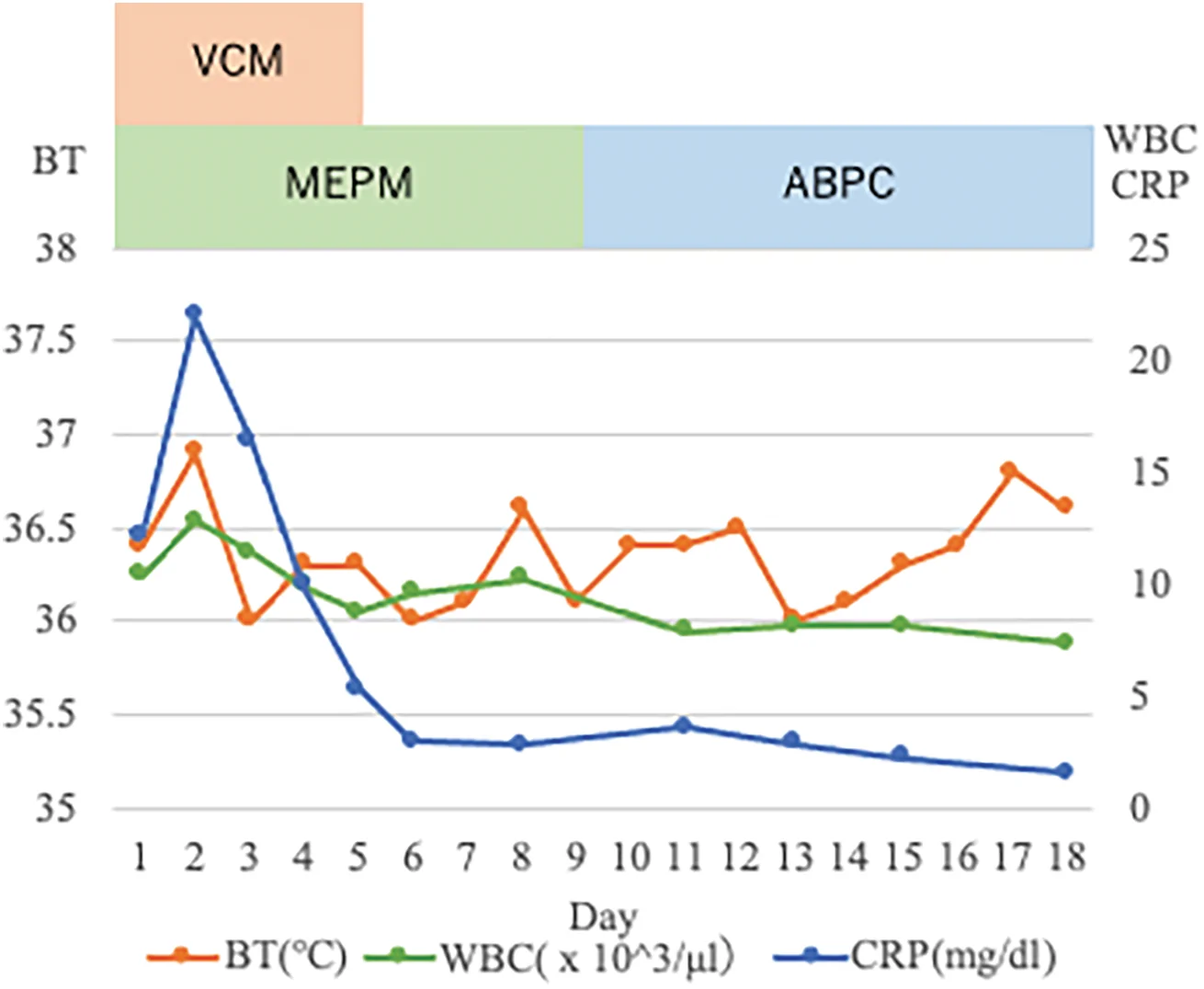
Clinical course until day 18. ABPC, ampicillin; MEPM, meropenem; VCM, vancomycin

The postoperative course was uneventful. The patient was transferred to a local hospital on POD 20. Intravenous antibiotic therapy was continued for 6 weeks, followed by oral trimethoprim–sulfamethoxazole after discharge (**Fig. 3**). No recurrence of infection was observed during the 2-year follow-up period.

## DISCUSSION

Infectious aortic aneurysms caused by *S. agalactiae* are extremely rare, and only a limited number of cases have been reported. A PubMed search using the keywords “*Streptococcus agalactiae*,” “infectious aortic aneurysm,” and “mycotic aneurysm” identified 17 previously reported cases.^4–20)^ **Table 1** summarizes the clinical characteristics of the previously reported cases and the present case. The lesions were evenly distributed between the thoracic and abdominal aorta, with 9 cases in each location. Most lesions had a saccular configuration; 17 of the 18 cases were described as saccular aneurysms.

**Table 1:** Clinical characteristics of 18 previously reported cases of infectious aortic aneurysm caused by *Streptococcus agalactiae*

Author (Year)	Age/Sex	Location	Max diameter (mm)	Risk factors	Surgical treatment	IV antibiotics (weeks)	Oral antibiotics (months)	Follow-up (months)	Outcome
Present case	77/F	Ascending aorta	65	Diabetes	Aortic replacement using plastic graft pre-soaked in Rifampicin	6	Ongoing	19	No recurrence
Derish et al. (2025)^4)^	72/F	Brachiocephalic artery	70 × 55	Diabetes	Aortic replacement	6	None	3	No recurrence
Liddy et al. (2023)^12)^	60/M	Aortic arch	33 × 27	Diabetes Remote GBS metatarsal osteomyelitis	Hybrid operation (Arch vessel translocation + TEVAR)	NA	NA	NA	NA
Segers et al. (2021)^7)^	69/M	Aortic root	40 × 25	Infectious endocarditis	Root repair with pericardial patch	NA	NA	NA	NA
Yan et al. (2021)^9)^	70/F	Abdominal aorta	36 × 30	Diabetes	Aortic repair using cryopreserved venous allograft	6	12	6	No recurrence
Valenti et al. (2021)^8)^	67/M	Abdominal aorta	41	None	Aortic replacement with homograft	NA	NA	3	No recurrence
Upapan et al. (2016)^10)^	66/M	Abdominal aorta	51 × 37	NA	Hybrid operation (Aortic replacement + EVAR)	2	Ongoing	20	No recurrence
Matsuda et al. (2014)^5)^	78/M	Aortic arch	100 × 80	Diabetes	None	NA	NA	NA	Death
Ledochowski et al. (2014)^11)^	86/M	Abdominal aorta	40 × 34	Steroid and Immunosuppressants user	Hybrid operation (Femoral to femoral bypass + EVAR)	NA	NA	1	No recurrence
Cozijnsen et al. (2013)^20)^	66/M	Thoracoabdominal aorta	39	Chronic alcoholism	Aortic replacement using plastic graft	2	NA	36	No recurrence
Thawait et al. (2012)^6)^	75/M	Abdominal aorta	57 × 40	None	Aortic replacement using plastic graft pre-soaked in Rifampicin	6	Ongoing	12	No recurrence
Masuhara et al. (2010)^13)^	59/F	Descending aorta	NA	Diabetes, Chronic alcoholism	Patch plasty using ePTFE	NA	NA	1	No recurrence
Yamamoto et al. (2009)^14)^	50/M	Abdominal aorta	NA	None	Aortic replacement using an equine pericardial roll	NA	NA	48	No recurrence
					Omental wrapping				
Chandrikakumari et al. (2008)^15)^	69/M	Abdominal aorta	NA	Diabetes, Chronic alcoholism	Aortic allograft replacement	NA	6	22	No recurrence
Iwaki et al. (2006)^16)^	59/M	Aortic arch	NA	NA	Aortic replacement using plastic graft	NA	NA	22	No recurrence
					Frozen elephant trunk				
Andreasen et al. (2002)^17)^	40/M	Abdominal aorta	40	None	Aortic replacement using plastic graft	4	Ongoing	9	No recurrence
Akashi et al. (2000)^18)^	61/F	Ascending aorta	50 × 40	After chemotherapy	Aortic replacement using plastic graft	NA	NA	16	No recurrence
Blackett et al. (1989)^19)^	61/M	Abdominal aorta	40	None	Aortic replacement using plastic graft	8	2	36	No recurrence
					Omental wrapping				

EVAR, endovascular aortic repair; F, female; IV, intravenous; M, male; NA, not available; ePTFE, expanded polytetrafluoroethylene; TEVAR, thoracic endovascular aortic repair

A clinically important finding across these cases was the potential for aneurysm enlargement despite antimicrobial therapy. Overall, aneurysm progression during antibiotic treatment was observed in 9 of the 18 cases, including the present case. In our patient, the pseudoaneurysm enlarged rapidly to 65 mm within 5 days after initiation of antibiotic therapy. Similarly, Matsuda et al. reported a thoracic aortic aneurysm that enlarged from 40 to 100 mm within 1 week despite antibiotic treatment and subsequently ruptured.^5)^ Although no definite preexisting aneurysm or ulcer-like projection was identified on preoperative imaging, severe calcific atherosclerosis of the ascending aorta may have provided a nidus for *S. agalactiae* seeding during bacteremia. Previous reports have suggested that bacteremic infection of an atheromatous plaque and atherosclerosis-related intimal disruption can compromise the normally infection-resistant aortic intima, leading to wall destruction and subsequent pseudoaneurysm formation.^21)^ In addition to these local aortic factors, advanced age, diabetes, and chronic alcoholism have frequently been reported as risk factors for infectious aortic aneurysms.^22)^ In our patient, poorly controlled diabetes (hemoglobin A1c level of 13.8%) may have contributed to rapid disease progression by promoting invasive infection and vascular wall destruction. Accordingly, in patients with *S. agalactiae* infection who have these risk factors, early imaging evaluation and short-interval CT follow-up should be considered.

With regard to surgical management, open surgical excision of the infected aneurysm with vascular reconstruction remains the cornerstone of treatment, as definitive source control requires complete removal of infected tissue and thorough debridement.^1)^ In this review, 17 of the 18 reported patients underwent surgical treatment. Of these, hybrid repair was performed in 3 cases with anatomy unsuitable for conventional open surgery, whereas most patients underwent open resection of the infected lesion followed by vascular reconstruction. Reported reconstructive strategies have included standard prosthetic grafts, rifampicin-soaked prosthetic grafts, homografts, and adjunctive omental coverage.^6,9)^ Omental flap coverage may provide additional benefit by obliterating dead space and enhancing local infection control. In patients with infectious thoracic aortic aneurysms, Yamashiro et al. reported better 5-year event-free survival with omental wrapping than without it.^23)^ In the present case, however, omental flap coverage was not used because of concern for concomitant pancreatic malignancy. The choice of reconstruction should be guided by the extent of infection, anatomic considerations, and available surgical resources.

Notably, no aneurysm recurrence has been reported among surgically treated cases during the available follow-up periods. In our patient, no recurrence was observed during 2 years of follow-up after prosthetic graft replacement and prolonged postoperative oral antibiotic therapy. Overall, the available reports suggest that favorable postoperative outcomes may be achieved with appropriate surgical intervention combined with antimicrobial therapy. However, because only a limited number of cases have been reported and the longest reported follow-up is approximately 4 years, long-term outcomes and the optimal reconstructive strategy remain uncertain. In addition, the optimal duration of postoperative suppressive antimicrobial therapy and the need for subsequent oral antimicrobial therapy remain unclear.

## CONCLUSIONS

Infectious aortic aneurysms caused by *S. agalactiae* are rare but may progress rapidly despite appropriate antibiotic therapy. Early imaging evaluation and timely surgical intervention should be considered when this diagnosis is suspected or confirmed, particularly in elderly patients and those with diabetes.

## DECLARATIONS

### Availability of data and materials

The datasets supporting the conclusions of this article are included within the article.
